# Dengue disease severity in Indonesian children: an evaluation of the World Health Organization classification system

**DOI:** 10.1186/1471-2334-7-22

**Published:** 2007-03-26

**Authors:** Tatty E Setiati, Albert TA Mairuhu, Penelopie Koraka, Mohamed Supriatna, Melvin R Mac Gillavry, Dees PM Brandjes, Albert DME Osterhaus, Jos WM van der Meer, Eric CM van Gorp, Augustinus Soemantri

**Affiliations:** 1 Department of Child Health, Dr. Kariadi Hospital, Semarang, Indonesia; 2 Department of Internal Medicine, Slotervaart Hospital, Amsterdam, the Netherlands; 3 Institute of Virology, Erasmus Medical Centre, Rotterdam, the Netherlands; 4 Dept. of Internal Medicine, University Medical Centre St. Radboud, Nijmegen, the Netherlands

## Abstract

**Background:**

Dengue disease severity is usually classified using criteria set up by the World Health Organization (WHO). We aimed to assess the diagnostic accuracy of the WHO classification system and modifications to this system, and evaluated their potential practical usefulness.

**Methods:**

Patients, admitted consecutively to the hospital with severe dengue, were classified using the WHO classification system and modifications to this system. Treating physicians were asked to classify patients immediately after discharge. We calculated the sensitivity of the various classification systems for the detection of shock and the agreement between the various classification systems and the treating physician's classification.

**Results:**

Of 152 patients with confirmed dengue, sixty-six (43%) had evidence of circulatory failure. The WHO classification system had a sensitivity of 86% (95%CI 76–94) for the detection of patients with shock. All modifications to the WHO classification system had a higher sensitivity than the WHO classification system (sensitivity ranging from 88% to 99%). The WHO classification system was in only modest agreement with the intuitive classification by treating physicians whereas several modified classification systems were in good agreement.

**Conclusion:**

The use of the WHO classification system to classify dengue disease severity is to be questioned, because it is not accurate in correctly classifying dengue disease severity and it lacks sufficient agreement with clinical practice.

## Background

Dengue virus infections are recognized as major public health problems in tropical and subtropical regions. Each year an estimated 100 million infections occur and between 250.000 and 500.000 severe cases are reported to the World Health Organization (WHO) [1]. Infection with any of the four dengue virus serotypes may give a broad spectrum of clinical manifestations ranging from no or minimal symptoms that cannot be easily distinguished clinically from other viral infections to a more severe form of disease that is characterized by a haemorrhagic tendency and increased vascular permeability. The WHO set up a classification system to differentiate between the self-limiting though debilitating dengue fever (DF), and the potentially lethal dengue haemorrhagic fever (DHF) [2]. According to these criteria, DHF is defined by the presence of fever, a haemorrhagic tendency, thrombocytopenia and some evidence of plasma leakage due to increased vascular permeability. DHF is further subdivided, with most severe cases categorized as dengue shock syndrome (DSS) when circulatory failure is present [2].

A standardized classification system for the severity of dengue virus infections is crucial for optimal communication of scientific data to improve our understanding of the pathogenesis and treatment of the disease. Incorrect disease severity classification may lead to faulty decision making in choosing the most appropriate treatment for the individual patient. Although the WHO classification system has been widely applied in research settings and publications, its use in everyday clinical practice has not proven easy or practical. In recent years, several studies reported difficulties with classification, inconsistencies in the WHO classification system and some found it necessary to define new categories to identify severe cases that do not meet the criteria for DHF or DSS [3-9].

These findings raise the question if the current WHO classification system is appropriate for the classification of dengue disease severity. To answer this, we assessed the diagnostic accuracy of the WHO classification system and modifications to this system. The presence of shock was used as marker of disease severity. By comparing the various classification systems with an intuitive classification done by treating physicians, we additionally evaluated the practical usefulness of the WHO classification system and the various modified classification systems.

## Methods

### Patients and clinical procedures

The study was conducted from February 2001 to April 2003 on the paediatric intensive care unit and paediatric ward of the Dr. Kariadi Hospital in Semarang, Central Java, a region in Indonesia where dengue is endemic. Patients, aged 2 to 14 years, consecutively admitted to the hospital with suspected severe dengue virus infection were included, provided that a parent or legal guardian gave informed consent. No strict criteria were used for inclusion in the study. Treating physicians could use the WHO case definition for dengue haemorrhagic fever as a guiding principle. If patients did not meet all criteria but a clinical suspicion of severe dengue virus infection was present, these patients were still eligible for inclusion. Members of the study team recorded demographic data, medical history, physical examination findings, clinical course and routine laboratory test results for each patient on a standard data form. The tourniquet test was performed on admission. Since it may be negative when circulatory failure is present the test was repeated after recovery from shock. Platelet count was performed daily. Haematocrit was measured at admission, every 2 hours for the first 6 hours and than every 6 hours until stable. Both haematocrit and platelet count were repeated in the event of clinical deterioration (i.e. recurrent shock, massive hemorrhage). Additional blood samples for diagnostic procedures were obtained on day of admission and on day 7 after enrolment. The ethics committee of the Dr. Kariadi Hospital approved all clinical and laboratory aspects of this study.

### Classification of disease severity

The study was initially designed to study pathophysiological mechanisms of hemorrhagic tendencies in patients with a severe dengue virus infection. For this we collected blood samples on several points in time during admission for the analysis of coagulation activity, fibrinolysis and inflammatory mediators. The study protocol and results of these studies have been described previously [10,11]. During the study, treating physicians were asked to classify patients as having DF, DHF or DSS immediately after patients were discharged. This intuitive classification was based on knowledge of medical history, physical examination findings, clinical course and all available results of routine tests (such as blood tests and chest X-ray). No structured algorithm was used to classify patients. The respective treating physicians were many different residents who worked under supervision of staff physicians at the paediatric intensive care unit and the paediatric ward of the Dr. Kariadi Hospital. Physicians were unaware of the fact that a structured classification was performed after completion of the study.

After completion of the study, two investigators (PK and ATAM) determined the presence of the following four clinical and laboratory manifestations on admission and during follow up in every patient using the standard data form: 1) fever or a history of acute fever, 2) haemorrhagic manifestations (at least a positive tourniquet test), 3) thrombocytopenia (platelet count ≤ 100.000 cells/mm^3^), and 4) signs of plasma leakage using laboratory findings (haematocrit ≥ 20% above average for age, haematocrit ≥ 20% compared to the haematocrit at hospital discharge, hypoproteinemia or hypoalbuminemia) and chest X-ray for the detection of pleural effusion. The presence of ascites on physical examination was not used as a sign of plasma leakage since routine physical examination has definite limitations in the precise diagnosis of ascites [12]. The determination of the presence of these manifestations was performed independently of each other and blind to the treating physician's classification. Disagreement was resolved by discussion and if necessary by adjudication of a third reviewer (E.C.M.v.G.).

Patients were subsequently classified as having DHF using these manifestations as proposed by the WHO [2]. If patients did not have one or more of these manifestations, they were considered to suffer from DF. In addition, several modifications to the WHO classification system were made as previously described [8]. Since all patients had fever or had a history of acute fever, the remaining three manifestations (haemorrhagic manifestations, thrombocytopenia and signs of leakage) were used to make the modifications. This resulted in six modified classification systems (Table 1). DSS was diagnosed when all DHF criteria were met plus evidence of circulatory failure defined as hypotension for age (systolic pressure <90 mmHg for those ≥ 5 years of age and <80 mmHg for those <5 years of age) or narrow pulse pressure (<20 mmHg) [2].

**Table 1:** Modified systems for the classification of dengue haemorrhagic fever.

Bleeding and thrombocytopenia
Bleeding and haemoconcentration
Haemoconcentration and thrombocytopenia
Bleeding and thrombocytopenia or haemoconcentration
Thrombocytopenia and haemoconcentration or bleeding
Haemoconcentration and bleeding or thrombocytopenia

### Diagnostic procedures

Paired blood samples were tested for serologic evidence of acute dengue infection. Commercially available enzyme-linked immunosorbent assays (Focus Technologies, Cypress, Calif., USA) were used for the detection of dengue virus specific IgG and IgM antibodies, according to the procedures described by the manufacturer. The sensitivity and specificity of these tests have been evaluated previously [13]. Cases were considered serologically-confirmed if the IgM ELISA was positive during the acute phase of disease (optical density of the sample higher than the optical density of the cut off serum provided by the manufacturer) and/or if a four-fold increase in IgG titre was demonstrated in paired acute and convalescent sera. For some patients, a definitive serodiagnosis was not possible because no convalescent sample was obtained. Detection of dengue virus antigen and/or viral RNA was attempted in these cases using a dot blot immunoassay and a dengue serotype specific reverse transcription PCR respectively [14]. Patients with definitive serodiagnosis and/or positive dot blot and/or positive PCR were considered to have confirmed dengue virus infection. The absence of dengue virus antigen, viral RNA and negative serology, was indicative of a non-dengue virus infection. When an alternative clinical diagnosis was absent and serology was inconclusive, patients were characterized as indeterminate.

### Statistical analysis

The evaluation of the diagnostic accuracy of the various classification systems was based on the presence of circulatory failure on admission and during follow up as the "reference standard". We calculated the proportion of patients with circulatory failure who were correctly classified as DHF (sensitivity). In addition, we calculated the proportion of patients classified as DF by the WHO classification system and without circulatory failure who were reclassified as having DHF when the modified classification systems were applied. The corresponding exact 95% confidence intervals (95% CI) were calculated from the binomial distribution.

As a measure of agreement between various classifications systems and the intuitive classification by the treating physician, we calculated the weighted kappa (κ_w_) statistic with a 95% confidence interval. The κ_w _values were interpreted as: poor agreement, ≤ 0.20; fair agreement, 0.21 to 0.40; moderate agreement, 0.41 to 0.60; good agreement, 0.61 to 0.80; or very good agreement, ≥ 0.81 [15]. A P-value ≤ 0.05 was considered to indicate statistical significance. Analyses were performed using SPSS 12.0.0. Agreement between the various classification systems and classification by treating physician was performed using MedCalc version 8.0.0.0.

### Role of funding source

The funding source had no involvement in the study design, the collection, analysis, and interpretation of data, in the writing of the report, and in the decision to submit the paper for publication.

## Results

A total of 198 patients were initially enrolled in this study. Parents from thirteen patients withdrew informed consent during follow up. Two patients appeared not to suffer from dengue during follow up (1 patient was diagnosed with measles and 1 patient was diagnosed with malaria). Of the remaining 183 patients, 28 patients (15%) had inconclusive serology and were therefore categorised as indeterminate. Three patients (2%) had definitive negative serology and were categorized as not dengue. The presence of dengue was objectively confirmed in 152 patients (83%): 115 by serology, 21 by dot blot immunoassay and 16 by dengue serotype specific reverse transcription PCR (seven Dengue virus serotype-1, four Dengue virus serotype-2, four Dengue virus serotype-3 and one Dengue virus serotype 1). Of the patients with confirmed dengue, 66 had evidence of circulatory failure: 52 (34%) had shock on admission (40 had hypotension, 2 had a narrow pulse pressure and 10 patients had both hypotension and a narrow pulse pressure) and 14 (9%) went on to develop shock within 48 hours after admission. Six patients (4%) died because of prolonged shock, massive haemorrhage and respiratory failure. Patient characteristics on admission are summarised in Table 2. All patients were previously well and had an unremarkable medical history.

**Table 2:** Baseline characteristics at admission of patients with confirmed acute dengue.*

Variable	All patients (n = 152)	Fatal cases (n = 6)
Age in years, median (IQR)	7 (5–10)	4 (4–6)
Male sex, n (%)	74 (49)	5 (83)
Duration of illness in days, median (IQR)	4 (3–4)	4 (4–5)
Haemorrhagic tendency, n (%) ¶	133 (88)	6 (100)
Positive tourniquet test, n (%)	103 (68)	3 (50)
Spontaneous haemorrhage, n (%) £	93 (61)	6 (100)
Hepatomegaly, n (%)	105 (69)	6 (100)
Systolic blood pressure in mmHg, median (IQR)	90 (80–100)	85 (68–93)
Hypotension for age, n (%) †	50 (33)	2 (33)
Pulse pressure <20 mmHg, n (%)	12 (8)	6 (100)
Pulse rate in beats/min, median (IQR)	120 (104–128)	130 (120–149)
Presence of pleural effusion, n (%) ‡	102 (67)	5 (83)
Haematocrit in %, median (IQR)	41 (36–45)	42 (35–47)
Platelet count in cells/mm^3^, median (IQR)	58.000 (37.000–85.000)	31.000 (27.000–76.000)
Platelet count ≤ 100.000 cells/mm^3^, n (%)	131 (86)	5 (83)

* n, denotes number; IQR, denotes interquartile range

¶ haemorrhagic tendency was present when at least one of the following was present: a) positive tourniquet test, b) petechiae, ecchymoses or purpura, c) bleeding from the mucosa, gastrointestinal tract, injection sites or other locations, d) haematemesis or melena

£ spontaneous haemorrhage was defined as all haemorrhagic manifestations not caused by a tourniquet test or venapuncture

† hypotension is defined to be a systolic pressure of <80 mmHg for those <5 years of age, or <90 mmHg for those ≥ 5 years [2]

‡ the presence of pleural effusion was assessed through a chest X-ray

### Diagnostic accuracy of the various classification systems

According to the WHO classification system, 20 (13%) patients were classified as having DF and 132 (87%) as having DHF. Of the DHF group, 57 (43%) could be classified as having DSS. Of the 66 patients with confirmed dengue and circulatory failure, 9 (14%) failed to meet all four criteria necessary for a diagnosis of DHF, and were thus classified as having DF. Six patients had a negative tourniquet test result and no bleeding manifestations during hospital admission, 1 patient never had a platelet count less than 100.000 cells/mm^3^, and the remaining 2 had no evidence of haemoconcentration or other signs of plasma leakage. The WHO classification system had a sensitivity of 86% (95%CI 76–94). The number of patients with circulatory failure classified as DHF changed when the WHO classification system was modified. Interestingly, all modifications had a higher sensitivity than the WHO classification system. The sensitivities of the various classification systems are shown in Table 3. Eleven patients without circulatory failure were classified as having DF according to the WHO classification system. Modification by the various systems came at the expense of some DF patients being reclassified as DHF as shown in Table 4.

**Table 3:** Sensitivity of the various disease classification systems in 152 patients with confirmed acute dengue virus infection.*

Criteria used for the classification of DHF patients	Number of patients with circulatory failure (n = 66) classified as DHF	Sensitivity (95% CI)
WHO classification system	57	86 (76–94)
Bleeding and thrombocytopenia	59	89 (79–96)
Bleeding and haemoconcentration	58	88 (78–95)
Haemoconcentration and Thrombocytopenia	63	96 (87–99)
Bleeding and thrombocytopenia or haemoconcentration	60	91 (81–97)
Thrombocytopenia and haemoconcentration or bleeding	65	99 (92–100)
Haemoconcentration and bleeding or thrombocytopenia	64	97 (89–100)

* n, denotes number; CI, denotes confidence interval; DF, denotes dengue fever; DHF, denotes dengue haemorrhagic fever; WHO, denotes World Health Organization.

**Table 4:** Proportion of patients without circulatory failure and classified as DF according to WHO criteria, that is reclassified to DHF when the various modified classification systems are applied.*

Criteria used for the classification of DHF patients	Number of DF patients without circulatory failure reclassified to DHF	Proportion (95% CI)
Bleeding and thrombocytopenia	5	45 (17–77)
Bleeding and haemoconcentration	1	9 (0–41)
Haemoconcentration and Thrombocytopenia	2	18 (2–52)
Bleeding and thrombocytopenia or haemoconcentration	6	55 (23–83)
Thrombocytopenia and haemoconcentration or bleeding	7	64 (31–89)
Haemoconcentration and Bleeding or thrombocytopenia	3	27 (6–61)

* DF, denotes dengue fever; DHF, denotes dengue haemorrhagic fever; WHO, denotes World Health Organization.

### Disease classification by treating physicians

Treating physicians classified 8 patients (5%) as having DF and the remaining 144 patients (95%) as having DHF. Of the 144 patients diagnosed as having DHF, 91 (63%) were considered to have circulatory failure at admission or at some point in time during admission in the hospital. In addition to the 66 patients with hypotension or narrow pulse pressure, treating physicians considered 25 patients with tachycardia, restlessness and cold and clammy skin as having compensated shock and subsequently diagnosed them as having circulatory failure. Agreements between the various classification systems and disease classification by treating physicians are shown in Table 5. The WHO classification system and three modified classification systems showed moderate agreement with classification by the treating physician: bleeding + thrombocytopenia, κ_w _value 0.53 (95% CI 0.41–0.64); bleeding + thrombocytopenia or haemoconcentration, κ_w _value 0.55 (95% CI 0.43–0.66); WHO classification system, κ_w _value 0.57 (95% CI 0.45–0.68); and bleeding + haemoconcentration, κ_w _value 0.59 (95% CI 0.47–0.71). The remaining three modified classification systems showed good agreement with classification by the treating physician: thrombocytopenia + bleeding or haemoconcentration, κ_w _value 0.64 (95% CI 0.53–0.74); thrombocytopenia + haemoconcentration, κ_w _value 0.67 (95% CI 0.56–0.78); and haemoconcentration + thrombocytopenia or bleeding, κ_w _value 0.70 (95% CI 0.59–0.81).

**Table 5:** Agreement between the various classification systems and classification by the treating physician.*

		Classification by treating physician		
		
		DSS	DHF	DF
WHO classification system	DSS	57	0	0
	DHF	23	52	0
	DF	11	1	8
Bleeding and thrombocytopenia	DSS	59	0	0
	DHF	23	52	5
	DF	9	1	3
Bleeding and haemoconcentration	DSS	58	0	0
	DHF	23	53	0
	DF	10	0	8
Haemoconcentration and thrombocytopenia	DSS	63	0	0
	DHF	25	52	0
	DF	3	1	8
Bleeding and thrombocytopenia or haemoconcentration	DSS	60	0	0
	DHF	23	53	5
	DF	8	0	3
Thrombocytopenia and haemoconcentration or bleeding	DSS	65	0	0
	DHF	25	52	5
	DF	1	1	3
Haemoconcentration and bleeding or thrombocytopenia	DSS	64	0	0
	DHF	25	53	0
	DF	2	0	8

* DF, denotes dengue fever; DHF, denotes dengue haemorrhagic fever; DSS, denotes dengue shock syndrome; WHO, denotes World Health Organization.

## Discussion

Our results show that a considerable number of dengue virus infected patients with circulatory failure is not identified correctly when the WHO classification system is applied. By modifying the combination of criteria that are included in the WHO classification system, we were able to identify more patients with circulatory failure. Overall, 86% of patients with circulatory failure were identified correctly by the strict WHO classification system. By contrast, four modified classification systems recognized more than 90% of patients with circulatory failure as DHF.

These findings are largely in line with observations made by Phuong and colleagues who studied a considerable larger group of Vietnamese patients [8]. They also demonstrated that all modified classification systems outperformed the WHO classification system in the identification of 631 patients with confirmed dengue and circulatory failure as a reference standard. A high sensitivity (> 90%) was found with the following modified classification systems: a haemorrhagic tendency with either thrombocytopenia or haemoconcentration (93%), haemoconcentration with either thrombocytopenia or a haemorrhagic tendency (94%) and thrombocytopenia with either haemoconcentration or a haemorrhagic tendency (95%). This comes at the expense of more patients being classified as DHF when any of the modified classification systems is used compared to the WHO classification system. However, if the purpose of classifying dengue disease severity is to identify the maximum number of patients with circulatory failure, then it would seem, that on the basis of Phuong's and our results, one of these modified classification systems is to be preferred.

Likewise, we found that the WHO classification system was in only modest agreement with the intuitive classification by treating physicians. Treating physicians were inclined to classify patients with evidence of plasma leakage as having DHF even in the absence of both thrombocytopenia and a haemorrhagic tendency. As a result, the modified classification systems haemoconcentration and thrombocytopenia, and haemoconcentration with either thrombocytopenia or a haemorrhagic tendency demonstrated good agreement with the intuitive classification by treating physicians. Phuong and colleagues noted that in clinical practice many physicians use the modified system of a haemorrhagic tendency usually together with thrombocytopenia rather than haemoconcentration. We showed that this does not hold true for our study setting. Treating physicians were inclined to classify severe cases as suffering from DHF using a combination of haemoconcentration with either thrombocytopenia or a haemorrhagic tendency instead of a haemorrhagic tendency together with thrombocytopenia.

Although several modified classification systems were in good agreement with the intuitive classification by treating physician, they still did not reach the magnitude of agreement we had expected (maximum κ_w _value 0.70). Two separate views of treating physicians played a prominent part in this. First, a considerable number of patients were classified as shock, although they did not meet the WHO criteria for shock. Treating physicians classified patients as having shock when symptoms and signs, such as cool and clammy skin, rapid pulse, decreased urinary output and confusion, indicating a stage I or compensated shock were present. This was done even in the absence of hypotension for age or a narrow pulse pressure. Second, patients who had no evidence of plasma leakage but did have a haemorrhagic tendency and thrombocytopenia were classified as DF. However, when circulatory failure was present a classification of DSS was given. To make a distinction between patients with and without evidence of plasma leakage an additional classification could be useful [4,5].

Several potential limitations of this study should be noted. First, grading of dengue disease severity was performed at the time of discharge or after completion of the study. By determining disease severity retrospectively, the classification systems may only serve a limited number of purposes. For example, they may be used to collect public health data on incidence of severe disease in different locations or over time. The classification systems are not suited for the identification of those at risk for developing severe disease, but they can be used as outcomes of interest in clinical trials and observational studies focusing on for example risk factors that may predict poor outcome. Second, patients' clinical course was assessed daily using standard data forms, regular haematocrit and thrombocyte measurements and additional diagnostic testing (i.e. chest radiography). This enabled us to collect all the data necessary for classification of disease severity. If regular laboratory testing or additional diagnostic tests are not performed because of for example limited resources, it may be difficult to demonstrate the presence of haemoconcentration with the possibility of misclassification. In addition, one can elaborate on the use of ultrasound in the detection of evidence of plasma leakage. We did not perform an ultrasound scan of the chest and/or abdomen because of practical issues. In several patients with circulatory failure, no evidence of plasma leakage was found despite the use of a chest radiography and frequent measurement of haematocrit (at admission, every 2 hours for the first 6 hours and than every 6 hours until stable). Whether ultrasound would have provided additional cases with evidence of plasma leakage, is uncertain but theoretically possible. Several studies reported on the use of ultrasound in determining the presence of pleural effusion, ascites or thickening of the gallbladder wall [16-19]. Although small amounts of abdominal and pericardial effusions can be detected using ultrasonography, its superiority over a combination of chest radiography and frequent measurement of haematocrit has not been demonstrated thus far. Finally, our analysis was confined to Javanese patients. As far as we know, only one other study performed in a Vietnamese population assessed the same modifications [8]. We can therefore only speculate on the possibility of extrapolating our findings to populations originating from other dengue affected areas like for example the American region.

An increasing number of dengue infections have been related to other unusual manifestations. These include fulminant liver failure, cardiomyopathy, ocular manifestations and neurological phenomena such as altered consciousness, convulsions, and coma resulting from encephalitis and encephalopathy [1,20-23]. Neurological manifestations were initially ascribed to complications secondary to DHF and DSS, although recently the invasion of the central nervous system by dengue viruses has been suggested [23]. Patients with symptoms or signs of a suspected infection of the central nervous system were not considered for inclusion in our study nor was additional diagnostic testing (i.e. lumbar puncture) done in included patients with minor neurological symptoms. We are therefore unable to pronounce upon the possibility of an increase in patients with encephalitis caused by dengue viruses. If additional studies show that such is the case than this would highlight the fact that the WHO classification needs revision.

## Conclusion

In conclusion, our results show that the WHO classification system is less accurate in correctly classifying dengue disease severity than all other modified classification systems. The implication of these findings is to question the use of the strict WHO classification system to classify dengue disease severity, all the more because it lacks sufficient agreement with how patients are classified in clinical practice. Additional research is needed in order to refine and improve the clinical usefulness of the system.

## Competing interests

The author(s) declare that they have no competing interests.

## Authors' contributions

TS, AM and PK wrote the first draft of the study protocol. DB, AO, JM, EG and AS contributed to the writing of the study protocol. TS, AM and MS were responsible for implementation of the study. TS and MS were responsible for management of patients, and data collection at the study site. PK and AO were responsible for all diagnostic procedures. AM and MM did all statistical analyses. TS, AM, and PK wrote the first draft of the report, and all authors read and approved the final manuscript.

## Pre-publication history

The pre-publication history for this paper can be accessed here:


